# Trends in the Use of Sphingosine 1 Phosphate in Age-Related Diseases: A Scientometric Research Study (1992-2020)

**DOI:** 10.1155/2021/4932974

**Published:** 2021-02-25

**Authors:** Qiong He, Gaofeng Ding, Mengyuan Zhang, Peng Nie, Jing Yang, Dong Liang, Jiaqi Bo, Yi Zhang, Yunfeng Liu

**Affiliations:** ^1^Department of Endocrinology, First Hospital of Shanxi Medical University, Taiyuan, 030001 Shanxi Province, China; ^2^Department of Second Medical College, Shanxi Medical University, Taiyuan, 030001 Shanxi Province, China; ^3^Department of Plastic Surgery, First Hospital of Shanxi Medical University, Taiyuan, 030001 Shanxi Province, China; ^4^Department of Radiotherapy, First Hospital of Shanxi Medical University, Taiyuan, 030001 Shanxi Province, China; ^5^Department of Pharmacology, Shanxi Medical University, Taiyuan, 030001 Shanxi Province, China; ^6^Key Laboratory of Cellular Physiology, Ministry of Education, Shanxi Medical University, Taiyuan, 030001 Shanxi Province, China

## Abstract

**Objectives:**

This study was designed to explore the intellectual landscape of research into the application of sphingosine 1 phosphate (S1P) in age-related diseases and to identify thematic development trends and research frontiers in this area.

**Methods:**

Scientometric research was conducted by analyzing bibliographic records retrieved from the Web of Science (WOS) Sci-Expanded Database dated between 1900 and 2020. Countries, institutions, authors, keyword occurrence analysis, and cooperation network analysis were performed using the CiteSpace and VOSviewer software.

**Results:**

A total of 348 valid records were included in the final dataset, and the number of publications and the frequency of citations have grown rapidly over the last ten years. The USA (*n* = 175), China (*n* = 42), and Germany (*n* = 37) were the three largest contributors to the global publications on S1P and aging, while the Medical University of South Carolina (*n* = 15), University of California, San Francisco (*n* = 13), and University of Toronto (*n* = 13) were the leading institutions in this field. Analysis showed that early studies primarily focused on the mechanism of S1P intervention in AD. While S1P and its relevant metabolites have remained a long-term active area of research, recent studies have focused more on interventions aimed at improving retinal degeneration, cardiomyopathy, multiple sclerosis, and diabetes, among others.

**Conclusions:**

It is worth mentioning that this manuscript is the first to describe any bibliometric analysis of S1P and its application in age-related interventions. This study includes a discussion of the (1) historical overview of the topic; (2) main contributors: journals, countries, institutes, funding agencies, and authors; (3) collaboration between institutes and authors; (4) research hot spots and zones; and 5) research trends and frontiers. This will enable scholars to understand the current status of S1P research in age-related diseases.

## 1. Introduction

Sphingosine 1 phosphate (S1P) is a derivative of sphingosine catalyzed by sphingosine kinases (SphKs) [1]. After coupling with S1P receptors (S1PRs), the SphKs/S1P/S1PRs axis regulates various cellular functions in the human body. Based on these interactions S1P has been shown to be associated with the genesis and development of multiple diseases, including diabetes, neoplasm, gastrointestinal diseases, neurodegenerative disease, kidney disease, and atherosclerosis [2–6]. Sadahira et al. [7] first described S1P as a strong inhibitor of cell motility in 1992 which was mediated by an unknown transmembrane protein. Recent advances in S1P suggest that it plays a much more important role in many age-related disorders than previously thought. Age-related diseases, such as Alzheimer's disease [8], multiple sclerosis [9], myocardiopathy [10], and diabetes [11], share similar pathogenesis, including changes in cell apoptosis, inflammation, and oxidative stress response. S1P has been shown to delay cellular senescence, maintain normal lipid metabolism, and alleviate hyperglycemia and inflammatory response [12–14]. This suggests that S1P has the potential to treat late-life illnesses.

As S1P continues to display desirable attributes for clinical applications so the number of studies evaluating this compound has continued to grow. However, this abundance of research has made identifying key messages more cumbersome, making it increasingly difficult for researchers and physicians to quickly find the key information they are interested in. In addition, there is still no comprehensive review of S1P applications in age-related diseases. Therefore, it is imperative that we analyze these critical hotspots, current knowledge gaps, and future trends in order to provide researchers with a more panoramic view of the subject. Scientometrics is an analysis technique used to identify key articles and themes using statistical and mathematical methods. Its superiority lies in evaluating the research quality and predicting the development trends rather than acting as a summary of the available data [15].

In this study, we used a bibliometric method to visually analyze the global status of S1P and age-related disease research. We hope to gain insights into the scope of this research by revealing the past and future research hotspots, which may encourage more significant research. In addition, this paper also summarizes the institutional and scientific leaders in this field with the hope of facilitating international academic communication and cooperation.

## 2. Methods

### 2.1. Data Collection

WOS is one of the world's largest and most comprehensive academic information resource libraries. Articles related to S1P and aging published between 1900 and June 26, 2020, were identified from the WOS Sci-Expanded Database. The search strategy was defined as TS (topic searches) = ((aging OR aged OR elderly OR senescence OR geriatrics OR gerontology OR geriatric OR geriatricians) AND (sphingosine 1 phosphate OR sphingosine 1-phosphate OR sphingosine-1-phosphate OR S1P OR S1P compound OR S1P receptor OR S1P receptors)). Topic searches include the title, abstract, author, and keywords. After excluding articles that were not related to aging (*n* = 10) or S1P (*n* = 2) or where the object of the study was botany (*n* = 2), we were left with 348 articles which were then included in the bibliometric analysis. The demographic information for each article including title, author name, year of publication, research institution, nationality, journal, abstract and keywords, article type, funding source, and citation density were recorded for further analysis.

### 2.2. Bibliometric Analysis

Scientometrics is a powerful analytical method that facilitates the quantitative evaluation of research distribution, trends, and collaborations between countries, institutions, and authors [15]. It is widely applied in all fields of science, including medicine [16, 17], chemistry [18, 19], mechanics [20, 21], computers [22], and economics [23]. In this study, the bibliometric analysis was completed using the CiteSpace (version 5.7.R1) and VOSviewer (version 1.6.13) software. Both of which converts the data from the publication into specific fields that can then be visualized as maps, which helps to uncover potentially hidden information subsumed by the sheer volume of data available. Our results reflect the number of documents produced by different authors and scientific institutions and their links [24, 25].

## 3. Results

### 3.1. Number of Published Articles and Their Citations

We identified a total of 348 studies describing S1P in aging. The earliest article was published in 1992 (*n* = 1), and the number of published manuscripts on this topic remained low for the next 16 years. Until 2008, we identified an exponential increase in the number of studies evaluating S1P. In addition, the *h*-index of the total publications was 48, where the *h*-index is an author-level metric designed to measure both the citation impact and productivity of a scientist [26]. A “successful scientist” would have an *h*-index of 20, an “outstanding scientist” would have an *h*-index of 40, and a “truly unique” individual would have an *h*-index of 60 [27], suggesting that most of the authors working within this field are relatively strong contributors.

**Figure 1:**
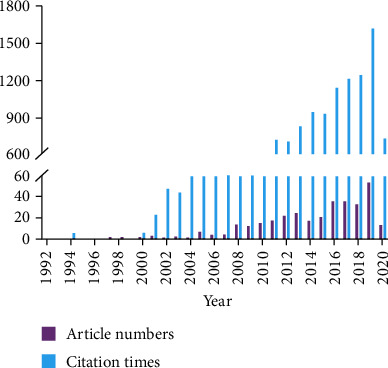
Number of publications and citations for S1P manuscripts produced between 1992 and 2020, per year.

These papers were cumulatively cited 11,573 times with the first citation appearing in 1993 (*n* = 2), followed by continuous growth. The total citation number reached its peak at 1,622 times in 2019.  Figure 1 shows the numbers of publications and citations for the manuscripts published between 1992 and 2020.

### 3.2. The Basic Composition of These Manuscripts

**Figure 2:**
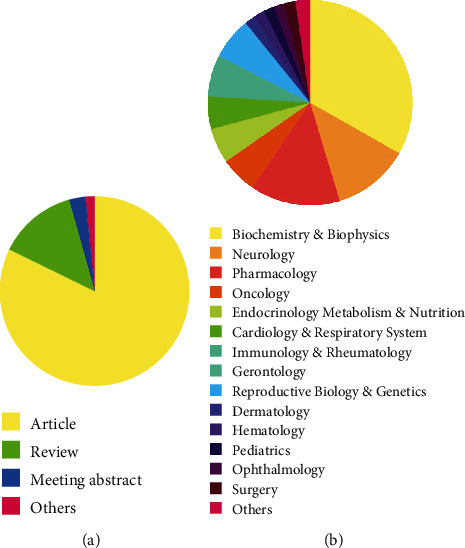
(a) Distribution of article types. (b) Distribution of research areas.

These 348 manuscripts were published in three languages: English (*n* = 346), German (*n* = 1), and Spanish (*n* = 1), and there were 287 articles, 46 reviews, 10 meeting abstracts, and 5 undefined publications within this portion of the literature.  Figure 2(a) describes the four article types in S1P and aging research. In addition, the research areas can be divided into approximately 15 groups: biochemistry and biophysics (*n* = 156), neurology (*n* = 57), pharmacology (*n* = 67), oncology (*n* = 27), endocrinology metabolism and nutrition (*n* = 26), cardiology and the respiratory system (*n* = 24), immunology and rheumatology (*n* = 19), gerontology (*n* = 12), reproductive biology and genetics (*n* = 31), dermatology (*n* = 8), hematology (*n* = 8), pediatrics (*n* = 8), ophthalmology (*n* = 7), surgery (*n* = 9), and others (*n* = 11), and Figure 2(b) describes the distribution of these 15 research areas.

### 3.3. Most Common Journals and Most Cited Articles

The top ten most common journals are listed in Table 1 in descending order by the number of published articles, with *Plos One* (*n* = 9), the *Journal of Biological Chemistry* (*n* = 7), and *Scientific Reports* (*n* = 7) being the three most popular journals.

Articles were sorted and listed in Table 2 in descending order of their total number of citations. The top 10 most cited articles were published between 2007 and 2018. There were two articles that have been cited more than 1000 times, published in *Nature Reviews Molecular Cell Biology* (impact factor (IF) = 43.35; 1884 citations) and the *New England Journal of Medicine* (IF=70.67; 1565 citations).

### 3.4. Countries, Institutions, and Funding Agencies

**Figure 3:**
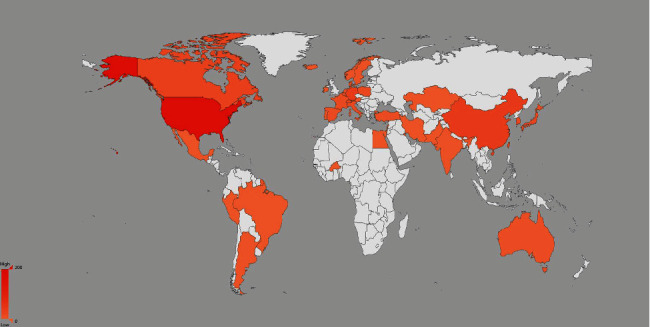
S1P and Aging publications per country.

The distribution analysis of the countries, institutions, and funding agencies implicated in these studies can provide valuable information on the academic environment surrounding this research topic. There were a total of 44 countries listed in the S1P and aging articles with the top 10 contributors including the USA (*n* = 175, 50.3%), China (*n* = 42, 12.1%), Germany (*n* = 37, 10.6%), Canada (*n* = 30, 8.6%), England (*n* = 24, 6.9%), Japan (*n* = 22, 6.3%), South Korea (*n* = 18, 5.2%), Italy (*n* = 17, 4.9%), Switzerland (*n* = 17, 4.9%), and Poland (*n* = 15, 4.3%).  Figure 3 shows the details of each country's contribution with deeper colors representing a higher density of publications.

**Figure 4:**
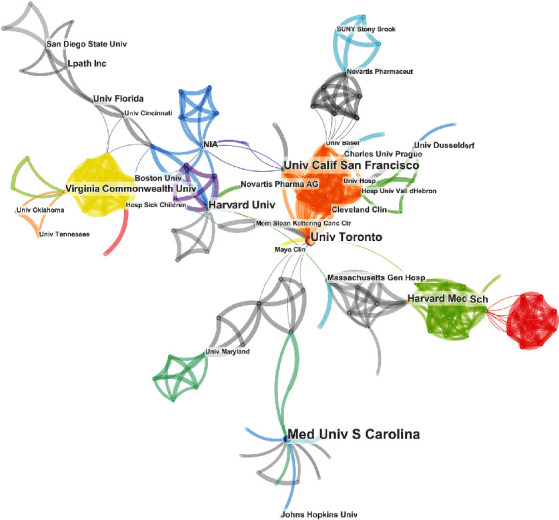
Institutions linked to S1P and its application in age-related research.

A total of 705 organizations have been linked to S1P articles focusing on its application in aging. The 10 most prolific organizations are as follows: Medical University of South Carolina (*n* = 15, USA), University of California, San Francisco (*n* = 13, USA), University of Toronto (*n* = 13, Canada), Virginia Commonwealth University (*n* = 9, USA), Harvard University (*n* = 8, USA), Harvard Medical School (*n* = 7, USA), Stony Brook University (*n* = 7, USA), University of Kentucky (*n* = 7, USA), Johns Hopkins University (*n* = 6, USA), Mayo Clinic (*n* = 6, USA), Novartis Pharma Schweiz AG (*n* = 6, Switzerland), and the University of Alberta (*n* = 6, Alberta).  Figure 4 shows the major institutions and their connections; the bigger the name, the more the institution contributed; the thicker the link, the stronger the relationship between them.

At least 606 funding agencies have provided financial support for research into S1P and an aging, with Table 3 summarizing the number of articles supported by the top 10 funding agencies. Five US funders ranked in the top 10 funding agencies, providing financial assistance to 247 articles, which account for 71% of all the manuscripts evaluated in this study. China, Germany, Canada, Japan, and Switzerland were also significant supporters of these studies.

### 3.5. Author Analysis

**Figure 5:**
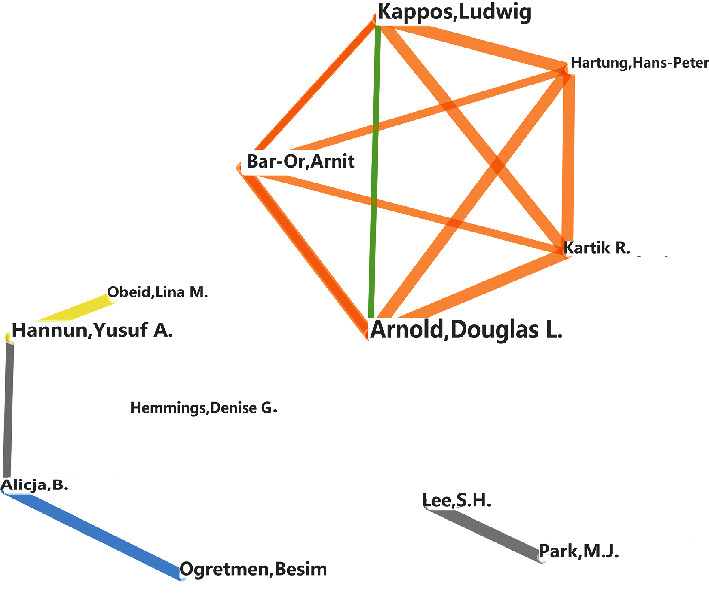
Cooperation network describing the relationships between the most productive researchers in S1P and age-related investigations.

These 348 articles were written by 2,347 authors, but only ten authors were credited on more than four articles on this subject, and more than half of these authors were from the USA. These most prolific authors also all share close relationships, for instance, the top two authors, Y.A Hannun and L.M Obeid, Lina M., come from the same university.  Figure 5 describes the links between the most prolific authors, with larger names indicating a greater contribution and thicker lines signaling stronger collaboration.  Table 4 describes the top ten most productive authors, their institutional affiliations, and country of residence.

### 3.6. Research Hot Spots and Zones

**Figure 6:**
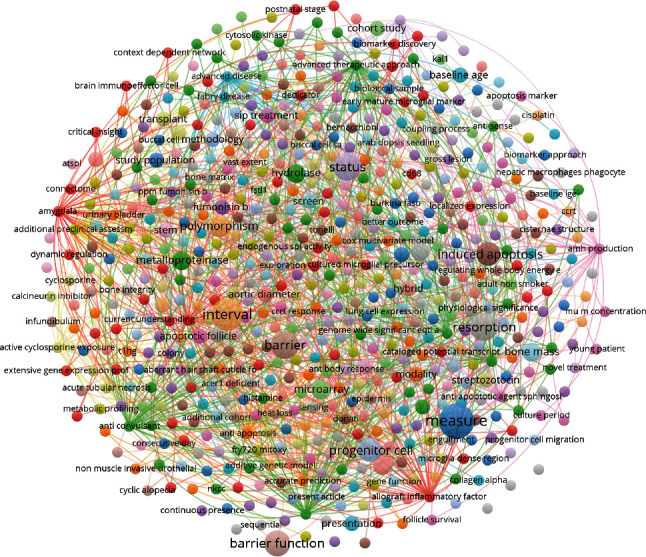
Keywords map in S1P and age-related researches.

Keywords are used to index articles and can be used to evaluate their major themes. Here, there were a significant number of keywords identified, but following a vocabulary frequency analysis, we were able to identify the most common keywords in S1P research (Figure 6). The larger the font in the figure, the higher the frequency of the keyword is; “acute tubular necrosis”, “induced apoptosis”, “barrier function”, “gene function”, “antibody respond”, and “resorption” were identified as the major pathological processes linked to S1P in aging. While “progenitor” and “brain immune effector cells” were the most common cell types. Several papers also referenced “hydrolase”, “metalloproteinase”, “collagen alpha”, “histamine”, and “streptozotocin” as potential targets.

**Figure 7:**
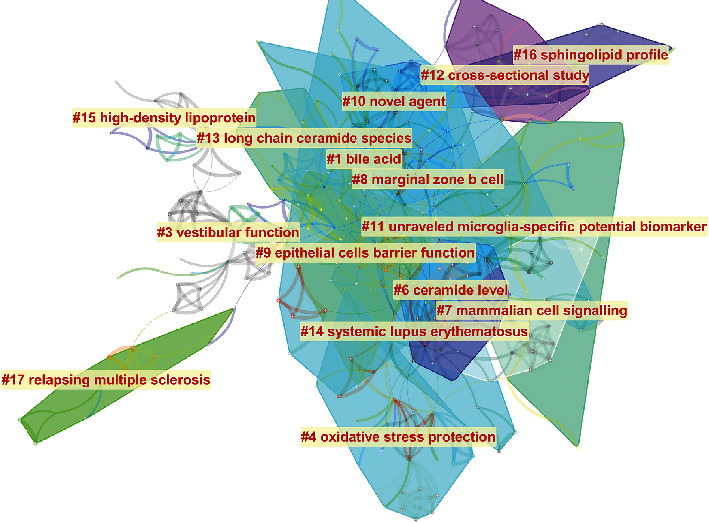
Keywords cluster map describing the hot spots in S1P age-related research.

Although there were several keywords, cluster analysis was still able to help us identify key research modules (Figure 7). Following data curation to remove duplicates this analysis revealed 15 clusters including sphingolipid profile, cress-sectional studies, novel agent evaluations, high-density lipoprotein, long-chain ceramide species, bile acids, marginal zone B cells, vestibular function, potential biomarkers for unraveled microglia, epithelial cell barrier function, ceramide level, mammalian cell signaling, systemic lupus erythematosus, relapsing multiple sclerosis, and oxidative stress protection. The overlapping portions of the image indicate where studies could be classified within more than one cluster.

### 3.7. Research Trends and Frontiers

**Figure 8:**
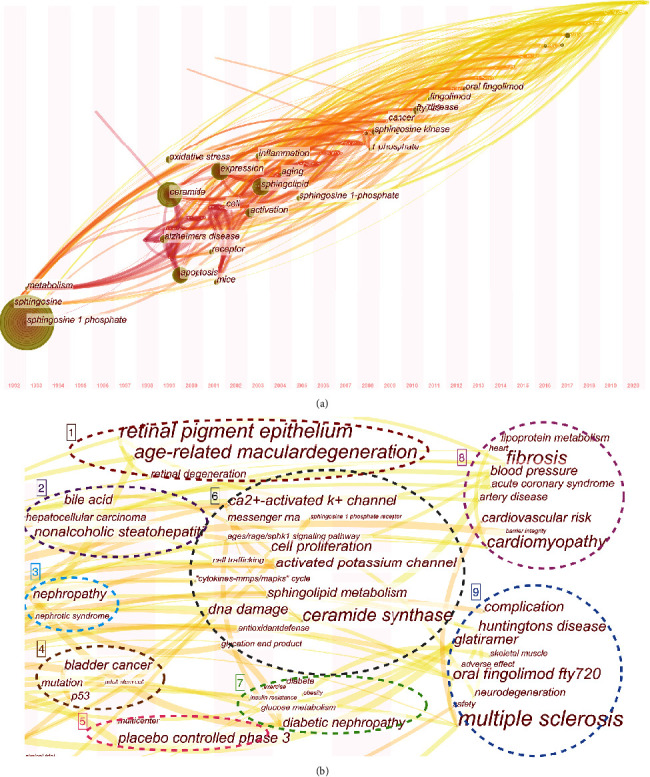
(a) Timeline view of common keywords. (b) Focused visualization of popular keywords used between 2016 and 2020.

When the keywords were analyzed in chronological order, we were able to identify several trends within the field over the last 3 decades (Figure 8(a)). Before 2008, Alzheimer's disease and cellular metabolism, apoptosis, inflammation, and oxidative stress associated with Alzheimer's disease were the key focus of this kind of research. Then, between 2009 and 2015, we see the emergence of cancer, sphingosine kinase, and fingolimod as novel research interests within this field. It is worth noting that S1P and its relevant metabolites have been the largest area of research throughout.

To further identify the research frontiers, we narrowed the timeline and evaluated the most common keywords applied to papers produced between 2016 and 2020 (Figure 8(b)). This analysis revealed nine emerging fields of research, which could be classified as research into retinal disease, digestive system diseases, nephrosis, cancer, clinical research methods, biochemistry, diabetes, cardiovascular system diseases, and nervous system diseases. Research into retinal disease, cardiovascular system diseases, and nervous system diseases were more common than the others suggesting that these topics are emerging focus areas in this field. When referencing either Figures 8(a)or 8(b), the larger the item, the higher the frequency of those keywords; the thicker the link, the stronger the relationship between them.

### 3.8. Research into Diabetes

**Figure 9:**
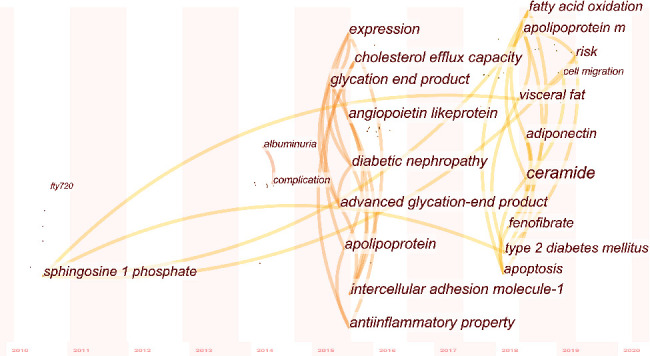
Timeline view of the keywords in diabetes from 2010 to 2020.

Given the interest in evaluating S1P, aging, and diabetes, we went on to complete a more focused keyword analysis of these manuscripts.  Figure 9 shows the timeline view of the different keywords used in diabetes and age-related S1P research. The first study in this field was published in 2010 and focused on FTY720 (Fingolimod). After three years with no additional publications on this topic, there was a sudden increase in available literature between 2014 and 2020. Between 2016 and 2017, diabetic nephropathy was the primary focus of these studies with angiopoietin-like protein, apolipoprotein, intercellular adhesion molecule-1, and glycation end products being the primary effectors under evaluation. In addition, bioprocesses such as cell migration, apoptosis, and fatty acid oxidation have drawn increasing attention from scholars evaluating S1P and aging-related diabetes in recent years.

## 4. Discussion

### 4.1. Developing Trends in S1P and Aging

In this study, bibliometrics and visual analysis were used to map the structure and evolution of S1P research and its application in age-related diseases. By combining the keyword clusters (Figure 7) and timeline view of the main keywords (Figure 8), we were able to show the expansion of this topic from only Alzheimer's disease to the inclusion of several other age-related disorders including macular degeneration, hepatocellular carcinoma, nephropathy, bladder cancer, diabetes, acute coronary syndrome, multiple sclerosis, and Huntington's disease. In addition, this analysis revealed the deepening molecular understanding of this compound and its underlying mechanisms from targeted evaluations of oxidative stress and inflammatory responses to the evaluation of gene signaling pathways, messenger RNAs, DNA damage, and a complex understanding of the age/rage/Sphk1 signaling pathway. However, sphingolipid metabolism and ceramide synthase have remained an active research area throughout this period.

When we narrowed the range of analysis down to the aging, S1P, and diabetes, we were able to identify only eight publications in the WOS Sci-Expanded Database as of June 26, 2020 (Figure 9). These studies focus on both diabetes and diabetic complications and emphasize the association between lipid and glucose metabolism. Apolipoprotein M, cholesterol efflux capacity, adiponectin, and intercellular adhesion molecule-1 have begun to emerge as key targets for researchers working in this field, and while diabetic nephropathy was the primary pathological focus for the last three years with compounds such as angiopoietin-like protein, glycation end products, and fenofibrate featuring heavily in these evaluations, we can see a recent trend toward the evaluation of bioprocesses such as cell migration, apoptosis, and fatty acid oxidation. Given the lack of data in this area, this may well prove to be the next hot topic in S1P and aging-related research and thus may be of interest to key researchers in the field.

### 4.2. Research Implications

The bibliometric analysis employed in this study has some implications for both clinical practice and academic research.

First, the leading research groups, with the greatest number of publications and authors, are all located in the USA. Given this, researchers around the world should try to find ways to cooperate and communicate with these top institutions in the future in order to advance this field in more diverse populations. The University of California, San Francisco, and the University of Toronto work closely on S1P and age-related research (Figure 4) and were tied for second place in the number of publications produced. Similarly, there is a close academic relationship between Hannun and Obeid (Figure 5), which may help to explain how they became the most published authors in this area of research. It should be noted that the close association between these institutions and authors may boost their research achievements and outputs.

Second, scientific practitioners need to pay more attention to the concentration of the S1P axis. With the accumulation of research, we have a deeper and more comprehensive understanding of S1P. In the early stages of research S1P, a newly recognized lysophospholipid was thought to be responsible for neuronal vulnerability to oxidative stress [28]. However, recent studies have confirmed that S1P functions to prevent the loss of synaptic plasticity, cell death, and neurodegeneration, which is beneficial for maintaining neuronal health [29, 30]. The reason behind these seemingly contradictory phenomena may be “sphingolipid rheostat” which suggests that it is the relative concentration of S1P instead of the compound itself that determines its effect in cellular systems [31]. The sphingolipid metabolites ceramide, sphingosine, and S1P constitute the S1P axis, but these metabolites are interconvertible and usually perform different functions. Active S1P stimulates growth and suppresses apoptosis [32], while ceramide and sphingosine usually inhibit proliferation and promote apoptosis [33–35]. Therefore, when studying S1P and diseases caused by aging, researchers should not only evaluate the effects of S1P but should also monitor the concentration of S1P in the circulation and the cumulative content of its byproducts and interaction partners.

Third, S1P has the potential to treat age-related diseases. As a new agent, S1P may reduce the side effects caused by biological incompatibility because it is an endogenous lipid molecule naturally found in the human body. FTY720 (Fingolimod) is the first Food and Drug Administration- (FDA-) approved S1P-related drug. It is designed to treat multiple sclerosis, which inhibits lymphocyte egress from lymphoid tissues by downregulating S1PR [36]. So far, the top three highest IF articles on S1P in age-related diseases are all about clinical drug trials. They are *A Placebo-Controlled Trial of Oral Fingolimod in Relapsing Multiple Sclerosis* (IF=70.67), *Oral fingolimod in primary progressive multiple sclerosis (INFORMS): a phase 3, randomised, double-blind, placebo-controlled trial* (IF=59.10), and *Siponimod* versus *placebo in secondary progressive multiple sclerosis (EXPAND): a double-blind, randomised, phase 3 study* (IF=59.10). So FTY720 has drawn a lot scientific attention on treating multiple sclerosis. However, S1P-related compounds have not yet been used to treat other late-life illnesses, such as diabetes, hypertension, and coronary heart diseases. International drug development in this field should be promoted and direct cooperation among hospitals, universities, and industries should be encouraged to accelerate the clinical testing of novel S1P products.

Fourth, the interconnections between age-related illnesses should receive some focus moving forward. Diseases caused by aging share common and interactive mechanisms. For example, hyperlipidemia is a risk factor for cardiovascular disease and changes in the lipid metabolism have also been linked to insulin resistance and neurodegeneration [37, 38]. Based on this, the study published in July 2020, which focuses on the function of S1P in both diabetes and neurological failures [39] may mark the beginning of a new era in S1P research. In addition, evaluating the interconnection and unintended side effects of S1P and age-related illnesses may reveal novel avenues for research in the future and broaden the clinical value of S1P.

### 4.3. Strength and Limitations

To our knowledge, this is the first study to systematically analyze the research related to S1P and aging-related illnesses using a bibliometric method. However, this study also has some limitations that should be addressed in the future. First, to meet the reference formatting requirements of the CiteSpace software, we could only retrieve articles from the WOS database. Future studies should be expanded to include studies from other databases such as PubMed if this technical limitation can be overcome. Second, as the research into S1P and aging is still in its early stages, there are only 348 articles included in this study, increases in the number of clinical evaluations and laboratory studies would of course enrich this type of analysis in the future.

## Figures and Tables

**Table 1:** Top 10 contributory journals.

Journal	Number of articles	IF (2019)
*Plos One*	9	2.77
*Journal of Biological Chemistry*	7	4.10
*Scientific Reports*	7	4.01
*Biochimica Et Biophysica Acta Molecular and Cell Biology of Lipids*	6	4.40
*Arteriosclerosis Thrombosis and Vascular Biology*	5	6.61
*International Journal of Molecular Sciences*	5	4.18
*Journal of Lipid Research*	5	4.74
*Lancet Neurology*	5	28.75
*Molecular Neurobiology*	5	4.58

**Table 2:** Top 10 most cited articles.

Title	Institutions	First author	Journal	Publication year	IF (2019)	Total citations
Principles of bioactive lipid signalling: lessons from sphingolipids	Medical University of South Carolina, USA	Hannun, Yusuf A.	*Nature Reviews Molecular Cell Biology*	2008	43.35	1884
A placebo-controlled trial of oral fingolimod in relapsing multiple sclerosis	University of Basel, Switzerland	Kappos, Ludwig	*New England Journal of Medicine*	2010	70.67	1565
Bioactive sphingolipids: metabolism and function	Medical University of South Carolina, USA	Bartke, Nana	*Journal of Lipid Research*	2009	4.74	368
Deregulation of sphingolipid metabolism in Alzheimer's disease	Mount Sinai School of Medicine, USA	He, Xingxuan	*Neurobiology of Aging*	2010	4.39	252
Sphingolipids in mammalian cell signalling	University of Manchester, UK	Ohanian, J	*Cellular and Molecular Life Sciences*	2001	7.01	183
Sphingolipids and cancer: ceramide and sphingosine-1-phosphate in the regulation of cell death and drug resistance	Medical University of South Carolina, USA	Ponnusamy, Suriyan	*Future Oncology*	2010	2.27	182
Sphingosine-1-phosphate produced by Sphingosine kinase 1 promotes breast cancer progression by stimulating angiogenesis and lymphangiogenesis	Virginia Commonwealth University School of Medicine, USA	Nagahashi, Masayuki	*Cancer Research*	2012	8.37	163
How to preserve fertility in young women exposed to chemotherapy? The role of GnRH agonist cotreatment in addition to cryopreservation of embrya, oocytes, or ovaries	Rambam Medical Center, Technion-Faculty of Medicine, Israel	Blumenfeld, Zeev	*Oncologist*	2007	5.25	162
Oral fingolimod in primary progressive multiple sclerosis (INFORMS): a phase 3, randomised, double-blind, placebo-controlled trial	Mount Sinai School of Medicine, USA, etc.	Lublin, Fred	*Lancet*	2016	59.10	156
Siponimod versus placebo in secondary progressive multiple sclerosis (EXPAND): a double-blind, randomised, phase 3 study	University of Basel, Switzerland, etc.	Kappos, Ludwig	*Lancet*	2018	59.10	150

**Table 3:** Top 10 S1P and its application in age-related research funders.

Funding agencies	Number of articles	Countries
United States Department of Health Human Services	105	USA
National Institutes of Health	104	USA
National Natural Science Foundation of China	18	China
NIH National Institute on Aging	17	USA
NIH National Cancer Institute	13	USA
German Research Foundation	12	Germany
Canadian Institutes of Health Research	10	Canada
Ministry of Education Culture Sports Science And Technology	8	Japan
Novartis	8	Switzerland
US Department of Veterans Affairs	8	USA

**Table 4:** Authors with more than 4 articles in S1P and age-related investigations.

Author	Number of articles	Affiliation	Country
Hannun, Yusuf A.	9	University of New York	USA
Obeid, Lina M.	8	University of New York	USA
Kappos, Ludwig	7	University of Basel	Switzerland
Arnold, Douglas L.	5	McGill University	Canada
Bar-Or, Arnit	5	University of Pennsylvania	USA
Hartung, Hans-Peter	5	Heinrich Heine University	Germany
Hemmings, Denise G.	5	University of Alberta	Canada
Lee, S. H.	5	University of Ulsan	Korea
Mao, Cungui	5	Stony Brook University	USA
Ogretmen, Besim	5	Medical University of South Carolina	USA

## Data Availability

The data supporting this scientometric research study are from previously reported studies and datasets. The processed data are available at Web of Science or from the corresponding author upon request.

## Abbreviations

FDA: Food and drug administration
FTY720: Fingolimod
IF: Impact factor
S1P: Sphingosine 1 phosphate
S1PR: Sphingosine 1 phosphate receptor
SphK: Sphingosine kinase
WOS: Web of science.
